# Situated Teacher Professionalism Understanding the Exploratory and Collaborative Aspects of Teacher’s Professionalism

**DOI:** 10.1007/s12124-025-09914-7

**Published:** 2025-06-10

**Authors:** Tilde Lykke Mardahl-Hansen, Charlotte Højholt

**Affiliations:** Department of People and Technology, Roskilde University, Roskilde, Denmark

**Keywords:** Situated professionalism, Teacher perspectives, Everyday school life, Participation possibilities, Situated inequality

## Abstract

The aim of this article is to contribute to understandings of teacher professionalism that address how teachers work with the dynamic interplay of everyday life as part of creating participatory possibilities for children and young people in concrete teaching situations. In doing so, the article contributes to the development of a ‘situated psychology’ with concepts that can strengthen the understanding of teacher professionalism as subjectively exploratory and collaborative. This is partly due to theoretical and political problems with dominant understandings of teacher professionalism, which fail to take account of the social complexity of everyday life in schools and are criticised for decontextualising and instrumentalising teacher professionalism, and partly to the authors’ concern with the unequal conditions of children’s participation in school learning communities. Recent research on ‘situated inequality’ points to a connection between children’s unequal possibilities to participate and manage in school and teachers’ professional conditions for working with children’s conditions to participate in school. Drawing on practice theory, a subject-scientific concept of ‘conduct of everyday life’, and Donald Schön’s theory of professionalism in practice, the article provides stepping stones for understanding teachers’ professionalism as situated in the everyday social life of the school, where teachers and pupils collaborate on differences and common tasks. The purpose is to strengthen the understanding of and conditions for teachers’ professionalism in working with children and young people’s conditions for participation in learning communities of school.

## Introduction

In this article we will unfold how a situated approach to understanding people’s everyday lives can contribute to an understanding of teacher professionalism. The reason for this is both a problem with dominant conceptions of professionalism that are not grounded in the everyday social practice of teaching, and a concern with the unequal conditions of children’s and young people’s participation and influence in leaning communities of school, a problem we have worked with as situated inequality.^1^

One point of developing a concept of situated teacher professionalism is thus also that we lack knowledge about how the development of pupils’ conditions for being together about school is linked to the development of teachers’ teaching practices.

This is based on an approach to children and young people as subjects who develop learning- and life opportunities by taking care of themselves, each other and their personal conduct of live in communities with others (e.g. Dreier, 2008; Højholt & Schraube, 2016; Holzkamp, 2013.) and an approach to their life contexts as dynamic social contexts organised as historical and complex practices (e.g. Lave, 2019; Juul Jensen, 2015; Axel, 2011, 2020). However, the concrete conditions for the common human task of developing conduct of life are unequal for children and young people, and they have different access to social resources and possibilities for action in relation to what they personally and together with others are trying to make work in their lives.

The last several years of theory development in relation to how people learn has focused on pupils’ participation in learning communities, but has the approach to the professionalism that addresses these both personal and social learning processes and their conditions kept up?

A commitment to children’s and young people’s unequal conditions for participating in and contributing to the social interactions, tasks and activities of school calls for an exploration of the professionalism aimed at developing conditions for different children’s learning through participation in school communities.

While an engagement with children’s unequal participation conditions in school can easily be linked to a preoccupation with something special and special interventions, here we are concerned with how ‘ordinary teaching professionalism’ can be characterised by continuously exploring different children’s different opportunities and creating possibilities for their participation in changing learning communities.

Teachers carry out dynamic developmental work on a daily basis and a key point of the article will be that this involves a flexible academic focus, continuous exploration of social situations and collaboration with both children and other adults. This challenges the dominance of technical rationality and the separation between reflection and action that Donald Schön problematised in his time, which is why we will also take up this discussion.

As we illustrate in the article, teachers have pointed out that current policy reforms of schools are based on a lack of recognition or misunderstanding of their professionalism and have led to more difficult conditions for bringing professionalism into play in school life. Research on children’s difficulties at school suggests that it is precisely when fixed and incomprehensible difficulties and categorisations of children are at stake that the general professionalism of teachers seems to be put out of play (Røn-Larsen, 2018).

We do not question that children and young people’s different life situations, experiences and resources give them different preconditions in their encounter with school, but we are concerned with how conditions for participation in school are not unambiguous and unchangeable, but mobile in social teaching situations. A concept of situated inequality invites us to consider unequal conditions of participation as something that is also created in processes characterised by professional powerlessness, unresolved conflicts and mutual abandonment between the parties involved. Such processes seem to bring fixed categorisations of learners into play and associated expectations that have implications for possibilities for participation (Højholt & Kousholt, 2018; Højholt & Schwartz, 2018; Tybjerg, 2023).

In this article, we deal with professionalism that concretely works with possibilities for participation and the development of learning communities in school teaching situations. The concept of situated inequality is merely an incentive and a conceptual background for the theoretical development of a situated understanding of teacher professionalism.

This attempt to focus on understandings of teacher professionalism should not be seen as another problem displacement in the direction of teachers’ individual professional competences, but rather as an attempt to help conceptualise and support a more situated pedagogy in the complexity of everyday life (Højholt & Mardahl-Hansen, 2021; Mardahl-Hansen, 2018a). In our experience, it can be essential to recognise and support the general professional work of teachers in difficult school situations. This requires theoretical conceptual work to understand what characterises this professionalism as it unfolds in everyday social practice.

The article thus focuses on how ‘education’ is not a singular entity but a dynamic and compound social practice. The communities and activities in which children and young people are expected to participate are in practice processual and conflictual—the content of shared activities and the academic and social conditions for participation in school are in constant flux. They are transformed by the concrete actions and interactions of teachers and pupils, not in a random way, but in relation to the ways in which schools are shaped by—and subject to—policy objectives and legal requirements, as well as institutional organisations, cultural practices and specific understandings and requirements of teachers’ professional tasks.

We will begin the theoretical discussions with a little empirical insight into how teachers themselves talk about their professional work, in order to use this curiosity to approach the various research proposals on professionalism, and why we find it relevant to ‘take up Schön’s legacy’ in an attempt to further develop concepts of situated professionalism. Next, we will try to unfold understandings of situated teacher professionalism in the social practice of the school by looking at a teacher’s handling of a teaching situation and argue how we find this illustrative of a particular teacher creativity. Through this, we arrive at a discussion of how the conditions for teachers to develop this professionalism in everyday school life have implications for the situated unequal conditions of children’s participation and have an everyday political significance.

## Teachers’ perspectives on their professionalism

At the beginning of the research project ‘Conflicts about children’s school life’ we started our empirical studies in two primary schools. The idea was to follow 4 teachers across tasks, classrooms and activities to investigate how teachers’ professional tasks appear in practice. This concern was part of a larger study of different parties’ perspectives on collaboration about inclusion in schools. It quickly became clear that teachers were linking problems in their everyday school life to problems in understandings of teacher professionalism.

Perhaps the clearest sign of this was the A3 poster of a school researcher with horns drawn on his forehead hanging in the teachers’ room. The poster was not personally aimed at the person depicted, but rather, we realised, at the way teachers perceived their professional tasks to be portrayed in the media and in political contexts with reference to research. Teachers perceived that political reforms and new demands and tasks contributed to difficult conditions for their work and that these were based on a lack of insight into everyday school life and the content of teachers’ professional tasks. Teachers’ criticism of research, illustrated by the poster in the teachers’ room, was linked to the political development of the school and wider conflicts about the content of teaching tasks.

This indicates how understandings of teacher professionalism are not neutral; they have a crucial impact on what we as a society allocate resources to and recognise as competent and relevant. It has also been problematised in the research literature that the very approach to the teaching task is a barrier to the development of teacher professionalism (e.g. Madsen, 2023).

In the dialogue with the teachers in the research project, it seems important for them to emphasise the social complexity of teaching tasks: *‘There are so many other things in a classroom besides the academic tasks*’. Failure to recognise the complexity of everyday life seems to be a major criticism of the research. It has increased our curiosity about how teachers’ professionalism relates to the development of children’s and young people’s social conditions for learning and the links between the academic and social aspects of the teaching task:

*‘As an educator, I must constantly consider how I can enable learning in this community? What kind of activities can the community handle*?’

Particularly in relation to the task of inclusion, there seems to be a discrepancy between teachers’ experiences in practice and the way they perceive the teaching tasks portrayed in the media, in courses and in policy statements. As a teacher in the project formulated:

*‘Inclusion is about all those little things that make a difference. Just giving a child a break, putting your hand on a child who is sad, noticing... laughing together, maybe giving a compliment to say I noticed you. Those little things we see and do. It’s just not what we hear about at conferences and courses on inclusion. There’s “the temperament barometer”, well-being measurements, “red-yellow-green”*^2^* and all sorts of systems’.*

In this quote, the teacher formulates a point that we recognise across research projects, that the development of possibilities for participation is created in social processes in everyday school life, and that the general pedagogical professionalism of teachers is often overridden when it comes to specific difficulties. The teachers’ perspectives provided an awareness of how their general pedagogical work is often understood relatively separately from understandings of what it means to create participation possibilities for children in difficulties.

Everyday knowledge and teachers situated pedagogical work seem to take a back seat in favour of a focus on special methods and technologies, measurement, preventive interventions and standardised professional procedures when we consider that “something special” is at stake for some children. The idea that inequality and vulnerability can be addressed through technical identification of students’ individual barriers to learning and participation in school is linked to dominant understandings of professionalism based on ideals of control, professional rationality and evidence (Biesta, 2017; Helsby, 1999).

## Technical Didactics

The educational researcher Per Fibæk Laursen describes current trends in the development of pedagogical practice in Danish primary schools since the 1990 s as ‘technical didactics’ (2020). The idea of ‘technical didactics’ stems from a tradition of educational research, often referred to as application-oriented research, whose development since the mid-twentieth century has involved a movement within educational school research where battles over standards and criteria for what constitutes ‘good knowledge’ have dominated the research field (Carr, 2006; Mardahl-Hansen, 2018a).

The dominant idea is that educational theory should be not only ‘theoretical’ but above all ‘practical’, in the sense that research should formulate rational principles for qualifying and making more effective the actions of teachers (ibid). Technical didactics is characterised by a preoccupation with questions of ‘effectiveness’ and ‘what works’ and has led, for example, to *‘ideals and demands for clear goals and learning goal-oriented teaching, testing of student learning, increased use of IT in teaching, evidence-based teaching methods, stronger school management, *etc.’ (Laursen, 2020, p. 279).

Technical didactics is based on the student as the object of effective learning strategies, and the teacher’s expertise is seen as closely linked to the application of predefined knowledge and manuals in solving problems in practice. In such an understanding, professionalism is fundamentally about being able to diagnose problems and solve defined problems with the recommended means (Lave, 2019, p. 115) to ensure maximum pedagogical functionality and adapted progression. A review of research on classroom management shows that different types of effectiveness studies currently dominate the field of school research, both nationally and internationally (Ministry of Education, 2014). This highlights a hierarchisation of knowledge forms, where abstract or exact knowledge (e.g. from tests) can outweigh knowledge from everyday life, for example when identifying interventions for children who are a concern.

Following the logic of technical didactics, much educational research tends to focus on teachers and teacher competencies as crucial for children’s learning and inclusion opportunities (e.g. Hattie, 2009; Nordenbo, 2008; OECD, 2011). However, Biesta, among others, points out that a linear understanding of the relationship between teacher competences and student learning is an expression of a decontextualisation and instrumentalisation of teacher professionalism and learning (for a more detailed critique, see Biesta, 2015; Priestley et al. 2015; Mardahl-Hansen, 2018a).

The focus of this article on understandings of teacher professionalism could perhaps also be read in this way, so we would emphasise that our focus is on teachers *as participants in social teaching practices*. It is a rejection of instrumental understandings of professionalism, in which social processes with and between pupils in the everyday life of the school take a back seat, and a concern that all those involved in and around the school set conditions for each other—and thus also for the teachers’ work with the conditions of interplay and participation of the pupils.

In particular, research on children, with a focus on schools, has highlighted the importance of ‘other children’ to children’s learning and development (Gulbrandsen, 1998; Rogoff, 2003; Valsiner, 1997; Stanek, 2014 and others). A concern with children’s access to social resources in school life and how they learn and develop agency together suggests that we need to explore how teachers work with the social conditions of participation. This means going beyond ideals of technical rationality and didactics to see professionalism in practice.

## The Reflective Practitioner

Almost half a century ago, Donald A. Schön offered a critique of technical rationality as the fundamental principle for how professionals think when they act (1983, 1987). His critical rejection of scholastic and instrumental approaches to professionalism formed the basis for efforts to identify concepts for alternative understandings of professionalism, with a focus on *professionalism in practice* (1983, 1987). Schön’s critique and conceptual development is therefore an obvious inspiration and theoretical springboard for the ambitions of this article to contribute to a conceptualisation of teacher professionalism that supports teachers’ situated work in developing the conditions for children and young people to learn in school.

According to Schön, professional practice is not characterised by problems that can be solved unambiguously or ideally, but by problematic situations characterised by uncertainty, disorder and relative indeterminacy. He was consequently concerned with developing new understandings and concepts of professionalism that reflect how professionals think and act in social, processual and dilemma-filled practices. Schön’s conceptual development was based on empirical studies of different types of professional practice, and although he did not deal specifically with discussions of teachers’ professionalism, he can be an inspiration (Bengtsson, 1995). Thus, we grasp Schön’s basic understanding that professionals (teachers) are subjects who reflect, create meaning and form new experiences as a starting point for action together with others. However, a focus on reflection is not necessarily sufficient or adequate for what it means to develop conditions for participation in school teaching practice.

Reflection is often understood as a mental act that implies a distance or detachment from the concrete, practical situation (Kirkegaard, 2016). As a result, Schön’s concepts have become widespread in a way that was not necessarily intended (Bengtsson, 1995) and the practice-based approach that was the starting point can be said to have receded over time in the use of Schön’s concept of reflection in professional education programmes. In this way, reflection in practice becomes an individual competence rather than a general basis for developing professionalism in social situations in specific institutional contexts. There is a need to both revitalise and develop Schön’s ideas on professionalism in practice (Kinsella, 2009).

In our view, it is crucial to maintain that the individual teacher’s ability to understand and act must always be understood in a concrete context. The development of teachers’ professional ways of thinking and acting cannot be understood only as linked to the individual teacher but must be understood as linked to their engagement in the professional teaching tasks in concrete social situations, and to collaboration with both pupils and other school parties.

Teachers’ development of action possibilities is not only about reflecting on possible solutions, but also about actively engaging in teaching practice as a complex and shared matter between teachers and pupils and other school parties, such as colleagues and parents. In the following, we will pick up the thread from, among others, Schön’s critical project to formulate understandings of professionalism in practice and to contribute to the development of a theory of situated teacher professionalism as a way to focus on *exploration and collaboration* as a central part of teachers’ professionalism.

## Introduction to The Example

We will now further develop the discussion on situated professionalism (see also Stanek et al., 2018; Røn Larsen & Stanek, 2024 for a discussion on situated professionalism) by including a practice example. The following example is from a participant observation conducted as part of the PhD project *‘An everyday perspective on primary school teachers’ work to create conditions for children’s participation and learning in primary school’*, where Mardahl-Hansen observed teachers’ everyday life in school across teaching, yard duty, preparation and various meetings—in and out of classrooms and tasks. The Ph.D. project was part of the larger research project ‘Conflicts about children’s school life’, which draws on traditions in practice research, where research processes are organized as collaborative processes between researchers and the professionals involved (Chimirri & Pedersen, 2019; Højholt, 2024; Kousholt & Højholt, 2022). The article’s attempt to contribute to a theory of situated teacher professionalism is thus based on collaboration with teachers on developing an understanding of the everyday tasks of teachers.

### A Teaching Situation

Pupils stream into the school gym, where Dorte, their teacher, is in her sports clothes and barefoot. One pupil has a note and needs to be excused from physical education (PE). Two pupils say they can’t do PE today because they have a headache and a bad knee respectively. A fourth has a painful wart on his foot. And what about the boy who has a bandage on his arm because of a sprain, but no note from home? Dorte listens to them all and tells them that those who don’t have a note from home should join in – that’s the school’s PE rule—and then they can judge how they are doing as they go along. The last pupils enter the sports hall after changing into sports clothes. Dorte asks some pupils who have kept their socks on to take them off, because it’s a school rule not to wear socks in the gym for safety reasons (you can slip). Two pupils present foot warts and are told that the ‘no socks rule’ does not apply to them and that they must keep their socks on. One without warts is unhappy about not being allowed to wear socks, but reluctantly takes them off anyway. After negotiating who will attend the class and what they will wear, the class can begin. As always, Dorte starts by asking the pupils to sit in the centre circle of the gym.

The first exercise of the day is presented according to Dorte’s planned programme. Dorte briefly and energetically explains what the ball game is about and goes through the rules. It’s a ball game similar to dodgeball, where the class is divided into two teams and many balls are thrown into the opponent’s half of the field in different ways. Dorte asks the pupils if anyone knows the game in advance? And do they understand what it’s about? Several pupils answer the question at the same time. Dorte says: ‘I only listen to those who sit in the circle and raise their hands without saying much beforehand’. Several of the pupils who wanted to be excused from PE before the lesson object to the game. Dorte makes some special rules—for example, you have to crawl on your knees at one point in the game, and the boy with the sore knee is allowed to play standing up. The girl with a headache is allowed to drop out if she thinks it’s getting too much during the game. Dorte divides teams by saying 1–2-1–2 alternately to the pupils in the circle. One boy tries to change seats, but Dorte insists that he must remain seated. Another asks if you can change teams. Dorte insists that the teams stay as she has made them. The game begins and the pupils seem engaged.^3^

### Contradictions and Dilemmas in Teaching Tasks

What we have described above is a selected *teaching situation*. Teaching situations are obviously a special type of social situation involving cooperation on educational activities in which the children and young people involved have different prerequisites for participating. What we particularly note in the example is how the teacher, Dorte, strives to develop and stabilise the teaching situation by (re)defining and (re)arranging the conditions for participation; Who can (not) participate? What should (not) be worn? What should pupils (not) engage in now? These immediate and tentative boundaries—rules, regulations and distinctions—both limit and support children’s possibilities to contribute to and collaborate on common teaching activities. The conditions for participation are not fixed but are evolving through efforts to create a common focus and at the same time to develop the common through continuous exploration of the different ways in which participants engage in the common activities. Attempts to organize social interaction involve authoritative determinations, evaluations and judgments as part of acting in a coordinated way in joint teaching activities. This emphasizes how the teachers’ position and tasks imply a special commitment to and preoccupation with subjects and students’ learning processes as a joint commitment.

At the same time, teachers need to be aware that arrangements for shared engagement and involvement in common classroom activities will necessarily have different meanings for pupils. When teachers organise social teaching situations, differences in possibilities for participation are both a starting point and at the same time processual and changeable, as the conditions for participation are continuously produced and reproduced through teachers’ and pupils’ actions, conflicts and attempts to arrange the common life (Busch-Jensen, 2015).

In what follows, we highlight this conflictual and dynamic coherence between common school tasks and situated possibilities for participation as essential for understanding teachers’ professionalism as linked to the connections between flexibility and common focus.

## Situated Learning and Flexible Development of Teaching Activities

It is a general and relatively banal point that children participate in school with different conditions. Children’s learning and participation in educational practices *are not the result* of teachers’ actions, children actively and subjectively relate to common activities from different social positions and with different perspectives, experiences and possibilities.

Understanding that teachers cannot control or predict students’ participation in teaching situations leads us to an increased curiosity about how teachers learn about different conditions for participation and how teachers collaborate with students to develop joint engagements and activities.

We want to highlight the *situated processes* in which Dorte assesses how it is relevant and meaningful to act in relation to the development of conditions for participation in this social teaching situation. Dorte’s ways of framing the conditions for pupils’ participation are created in a process of learning and making choices in a continuously evolving teaching situation.

For example, in a dialogue with Mardahl-Hansen about the specific physical education lesson, Dorte talks about having to deal with pupils who have different reasons for participating and who may be excluded from the planned academic activities:

Sports activities are something they [the pupils] must take part in. But there may be some pupils who think that taking part can make them vulnerable, and it shouldn’t. Some children may also think that sports activities can be violent. It’s a balancing act, they need to be involved, and they need to be challenged, but of course they have different boundaries that need to be respected. They must find a way in, otherwise it won’t work.

Dorte describes how teacher professionalism is linked to exploring and relating openly to the relationship between her pedagogical and didactic intentions with teaching and the pupils’ subjective and social conditions for participation. The formulation ‘the pupils must find their way’ can be understood to mean that teachers must continuously work in a concrete and situated way to investigate and support children and young people to engage in joint activities from their location in practice. *‘This is a very different ‘take’ on teaching than one that characterises teachers as leaders in control, setting the agenda for students, in ways intended to take precedence over students’ life projects’* (Lave, 2019, p. 98).

Pupils’ subjective perspectives on participation in social teaching activities are necessarily partly *unknown* to the teacher, and at the same time, (some) insight into or awareness of these is a crucial aspect of making teaching work. The inclusive practice of everyday life is, among other things, about examining what is at stake for different pupils in concrete situations. By listening, observing and asking, Dorte learns about the social conditions of the situation and develops new opportunities to take relevant action. In this way, there is a reciprocity at play between teachers and pupils in developing conditions for participation by transforming shared school life.

Teachers need to *decentre* their perspective to understand how teaching activities have different meaning for different pupils to flexibly focus, prioritize and support collaboration with and between pupils in relevant ways in concrete teaching situations (Axel & Højholt, 2019). Teachers often formulate this requirement as the need to ‘get to know’ their pupils. A knowledge they develop through personal experience and interaction with pupils and by sharing knowledge with other teachers and parents.

Dorte’s knowledge of the individual pupils and their social situation influences how she understands and responds to their ways of participating—for example, her knowledge of the pupils’ family situation and whether it is relevant to expect them to bring a note from home. This knowledge affects Dorte’s actions in the situation, but also how she chooses to follow up on the specific situation afterwards, for example by sharing knowledge with other teachers of the class.

This could potentially resemble an isolated perspective on specific children’s personal prerequisites for participation. It has been problematized elsewhere, for example, how low pedagogical expectations for children and young people in vulnerable positions can be part of marginalization processes in schools (Andersen, 2016; Kelly & Carbonaro, 2012). In Dorte’s formulations, however, we see how her understanding and development of individual pupils’ possibilities for participation is linked not only to knowledge of and attention to the individual pupil, but also to a curiosity about the specific teaching situation in which they are involved. Understanding teachers’ agency as closely linked to situated analyses and developments of social teaching situations naturally challenges the focus on individual competences and methods and points to an increased focus on teaching as an explorative, collaborative and transformative social practice. Dorte does not just respond to isolated needs of the children, but through insight into variations of specific concerns related to general matters of teaching, tinkering with the contradictions of school life.

## Reconciling Contradictory Concerns

Knowledge of children’s possibilities for participation relates both to knowledge of the individual pupil and to knowledge of the resources, social dynamics and ‘common causes’ of the learning community (Axel, 2020). Teachers need to understand and consider how the social interplay between children influence the development of a shared school life and, as part of this, consider the *content* of teaching activities as a condition for collaboration. The content of different school subjects is important—working with participation, interplay and learning communities is very different in e.g. German lessons, mathematics and physical education. Here about conflicts in physical education:

There are games and plays where there is too much physical contact and too many grey areas about the rules, and too many conflicts. Of course, they (the pupils) need to be challenged, but if you can’t talk through the conflicts with them, then there is no learning in those conflicts. Then it’s just conflict and we don’t get any further (Dorte, teacher).

The content of the common activities is part of Dorte’s exploration and commitment in relation to understanding and developing the conditions of participation for different pupils. During Dorte’s PE lessons, she must relate to many different aspects of specific sports activities, such as the competitive element, the need for cooperation in team sports, the room for diversity in participation methods, performance and assessment (PE is an examination subject), the desire for physical movement, and anxiety in the community can challenge the demands for body contact, etc. Teachers need to relate to the connections and contradictions of what pupils do together, as they reflect the composite and potentially conflicting considerations and tasks that characterise social teaching situations.

Schools are expected to take on large and often contradictory tasks in relation to the development of children and young people’s life chances and their participation in the development and continuation of society (Mardahl-Hansen, 2018a). There are continuous conflicts about all this, as many considerations need to be combined and prioritized. Professionalism seems to be related to connecting the different aspects of the common matter and thereby allowing conflicts to develop the teaching rather than becoming deadlocked (Busch-Jensen, 2015).

School problems are not an expression of mistakes, individual shortcomings or just seemingly endless problems for teachers to solve, but rather an expression of contradictions internal to the task of teaching – through dealing with these contradictions the teachers are developing their professional practice.

Dorte describes how to deal with the many different aspects of a teaching situation:

Social talk and conflict resolution is part of what we do. Part of our job is also to educate for democracy. That’s why they [the pupils] must learn, for example, how to verbalise conflicts. Both in pairs, or how many they are, but also in the larger forum [the class], because even if you are not directly involved in a conflict, it can be instructive. But it’s a balancing act, because it shouldn’t take up too much space. We must achieve the academic goals... thus in the classes where it takes up too much time, I have to say that we have to talk about it at other times than in class, for example during breaks.

Dorte explains how she has to ‘balance’ different aspects of her teaching practice, which are at the same time interrelated and challenge each other. The teaching task is constantly caught in dilemmas between many different considerations or ‘activity priorities’ (Højholt, 1996, p. 50; Mardahl-Hansen, 2018a, 2018b).

However, teaching practices are often understood as stable social systems in which disturbances appear to be external to the teaching task and individuals may appear to be the cause of disturbances to the social order. In such an understanding, teaching is potentially perceived as a standardized activity that can be performed ideally. We have addressed this as a dominant understanding of teacher professionalism as an expression of technical didactics, management and effectiveness.

However, if we examine teaching as a social practice, we see that teaching in practice is characterised by continuous, active efforts to (re)manage dilemmas and connections between different aspects of teaching situations. For example, in the quote above, Dorte talks about how she *sometimes* chooses to shut down a conflict between pupils to prioritise focus on the planned activities, and *other times* she prioritises resources to investigate and work with the content of a particular conflict. In a situated way, Dorte allows some aspects of school life to take a back seat and emphasises others—in a careful coordination of demands for academic progress and conflict resolution.

In this way, the dilemmas of prioritisation reflect a continuous need to make choices, prioritise resources and engage in processes, contradictions and understandings to make teaching work together. If the contradictions and conflicts become too irreconcilable, this can be perceived as a breakdown of teaching and lead to entrenched understandings of problems, with the risk of marginalising some pupils and neglecting some aspects of everyday school life (Waleng, 2024). In the words of Uffe Juul Jensen, creating social conditions for participation in education involves *‘a constant struggle to change circumstances that undermine our possibilities to develop or maintain common practices*’ (Juul Jensen, 1999, p. 94).

## Conduct of Everyday School Life

To develop a language for situated teacher professionalism we now include a concept of *conduct of everyday life* (Holzkamp, 2016). The concept of conduct of everyday life generally addresses the fact that it is through active engagement in social practices that people coordinate and connect different affairs in their lives. To pursue personal interests and meet the demands we face, we must actively seek to integrate, coordinate and prioritize between different aspects of our lives. In school, it can be about the need to combine academic engagement with access to social resources and taking care of oneself. The subjective necessity of creating connections in everyday life is formulated by Ole Dreier:

‘An everyday life with diverse demands and commitments may fall apart if it is not conducted so that it hangs together. Diverse and conflicting demands and commitments may pull it apart if she does not maintain a sufficient measure of coherence’ (Dreier, 2016, p. 22).

For both teachers and pupils (and other school stakeholders), managing school life involves active and subjective efforts to balance, combine, separate, develop, link and prioritise different aspects of school life that co-exist, are interrelated, potentially contradictory and conflictual. Conduct of everyday school life is thus linked to the development of possibilities of action through orienting in and influencing social processes, arrangements and activities in everyday school life.

Teachers’ conduct of everyday school life can also be expressed as teachers conduct of everyday *work* life in order to highlight the fact that they engage in a shared, everyday school life from a professional position as teachers with responsibility for teaching (and, as part of this task, children’s learning and well-being).

Paying attention to teachers’ conduct of everyday work life highlights the variety of tasks and relations that teachers are involved in. As observers we follow teachers in a diverse everyday work life—in and out of different teaching situations involving different subjects and classrooms, exchanges with colleagues, conversations with parents and perhaps counsellors or psychologists, preparation, network meetings, and so on. Some of the exploratory and collaborative work of teachers takes place across these activities. At the same time, by zooming in on the diversity of a single teaching situation, such as the start of a PE lesson, we have tried to illustrate a diversity of different considerations, aspects and demands that require teachers’ situated exploration and collaboration to carry out the teaching.

Professional practice involves a selective and professional commitment that requires, among other things, the courage to prioritise, to maintain, to demand and to insist. Teachers’ conduct of everyday work life develops both through necessity and through the development of preferences and understandings of relevance, as Dreier puts it:

She must develop more or less articulate and sustained stances about what is important, what she commits herself to and against and what she wants preserved or changed in the complex social practices she takes part in (Dreier, 2016, p. 23).

Teachers’ efforts to make teaching work necessarily involve priorities, questions of resource allocation and privilege some aspects of a teaching situation – and some school tasks—over others.

As a teacher you are part of a system - the school. You must meet the needs of the individual pupil and at the same time it is defined from above what we must do. It takes courage to dare to say, ‘I’m doing this because it’s necessary’ (Dorte, teacher).

Many parties—pupils, parents, school leaders, pedagogues and other resources persons and decision-makers—are involved in different ways in the development of school priorities and understanding of problems—and thus in the development of conditions for participation.

For example, teaching goals, curricula, regulations, division of labour and regulations of working hours are conditions for teachers’ possibilities to organise, integrate and conduct their daily work lives. This has implications for the professional possibilities to organise, integrate and construct daily life in such a way that different, and potentially conflicting, aspects of the teaching task can be handled in a coordinated way. Welfare state policies and objectives set the conditions for how teachers can explore the conditions of children’s participation and how they can act on the knowledge they continually gain through their engagement with concrete children and school classes. In this context, teachers can find themselves at the mercy of other’s ambitions and ideas—and torn apart by conflicting perspectives on their tasks. That said, working with many parties with different perspectives on children’s school life is part of teachers’ everyday lives and an important aspect of developing practice. In this collaboration, the parties involved compose the conditions for each other in a situated interplay, dealing with the differences, contradictions and possible conflicts.

A key point is that teachers work daily to develop conditions for participation and contribution in educational situations in collaboration with pupils, but the organisational and resource conditions for this can be extremely difficult.

The exploratory and collaborative aspects of teachers’ professionalism often seem to be under-recognised in understandings of teacher professionalism in policy frameworks for teachers’ professional work, and in inter-professional collaboration on children of concern. These aspects are further threatened by the drive for order, efficiency and the desire for harmony in social interactions.

## Exploratory and Collaborative Professionalism

As we have tried to illustrate and argue, teacher professionalism is both productive (creative) and processual and is closely linked to the development of relevant possibilities for the participation of different pupils in different teaching situations.

In order for teachers’ actions to have the intended meanings and for teaching practices to work for many different participants, teachers are quite dependent on collaboration with their pupils and the many parties involved in school. They also need to facilitate the collaboration between the pupils and between the parents. Conflicts in and around a school class are fundamental conditions for the practice of teacher professionalism and for working with the conditions of participation in learning communities.

Furthermore, this professionalism is based on a situated, everyday exploration of the dilemmas, contradictions and processes situated in the school, and also of the relevance and social meanings of different ways of dealing with them.

Current classroom dynamics, pupils’ modes of participation, interplay, as well as conflictual cooperation in the school (especially with and between parents) and also the winds of school policy are common features with which teachers have experience, but which are also subject to historical change and need to be continuously explored.

Doubt, curiosity and exploration that contribute to transcending, challenging or confirming teachers’ knowledge are central to developing relevant possibilities for action. In this sense, from a teacher’s perspective, developing the conditions for children’s and young people’s participation in school is closely linked to the exploration of situated interactions, processes and problem analysis (Mardahl-Hansen, 2021).

We have conceptualized such exploratory processes as “teachers’ everyday analyses” (Højholt & Mardahl-Hansen, 2021). These are based on teachers’ knowledge of the children involved, their experience, curiosity and exploration of connections across situations in school and children and young people’s other life contexts.

Teachers’ everyday analyses are thus an expression of a preoccupation with exploring and dealing with both the personal and social meanings of the ways in which school conflicts, dilemmas and contradictions are played out in everyday life at school in concrete situations and over time. Hence, the way we understand and promote this professionalism has implications for our possibilities to address the issue of inequality in schools.

## Teacher Professionalism and Situated Inequality in School

As mentioned, the article’s analyses of situated teacher professionalism are initiated, among other things, by a concept of situated inequality that highlights the importance of children’s and young people’s conditions for participating in and contributing to school learning communities. The teachers’ everyday analyses are therefore crucial for transcending marginalisation and segregation, because they hold potential knowledge about how school arrangements, processes, dilemmas and problems can present themselves as personal life challenges for some children.

Unequal possibilities for participation in schools are closely related to, among other things, the conflictual organisation of teaching situations, restricted opportunities for collaboration and the lack of possibilities for some children and young people to negotiate and change their position in the community and to influence the common conditions of the learning situations. The ‘possibilities of difference’ in schools (Madsen, 2023) are conditioned by teachers’ possibilities to develop teaching situations, teaching methods and expectations for participation in ways that enable relevant coherences in children’s lives and in teaching practice. This means (among other things) that teachers need to be both focused and guiding as well as exploratory and flexible in relation to the specific social conditions of possibilities.

Difficult conditions for these explorations of social processes and for collaboration with pupils to deal with the conflicts, dilemmas and contradictions in everyday school life matters for pupils’ opportunities to contribute and legitimacy to influence everyday school life. In this way, these issues also relate to the question of democracy in schools and the possibilities for school participants to develop agency.

There is a need to create good conditions for, strengthen and further develop teachers’ everyday analysis of the social conditions of children and young people. Not least because it is precisely through insight into the social processes and contexts of schools that we can recognise and work with situated inequality and exclusion in schools (Højholt & Mardahl-Hansen, 2021).

We are currently witnessing a greater willingness to consider practice in education, research and policy development (formulated, among other things, as a quest for ‘practicality’). Such a movement must take into account both Schön’s critical project and the criticism and further development of it—including the rejection of hierarchical divisions of knowledge, reductionism and simplifications, and the implementation of external ‘solution techniques’. To move beyond the tendency to isolate and individualise inequality in education, we need to think about teachers’ professionalism in new ways supporting exploratory and developmental work.

Following on from the illustrations of the many aspects of school life we find that the *conditions for reconciling* conflicting considerations—both related to the ‘internal contradictions of the cause’ and related to a diversity of different participants and perspectives on the school life—are important for the politics of everyday school life (see also Røn Larsen & Stanek, 2024). For example, politic of everyday life are about how the school develops, what it will be about—its content—and who can be part of its development and influence its everyday life. The political aspects refer to the distribution of resources, access to the social resources of school life, prioritisation and content in subjects and communities. Understandings of teacher professionalism and teachers’ societal tasks thus have implications for the situated inequality of everyday life.

## Data Availability

No datasets were generated or analysed during the current study.

## Footnotes

1 In other articles, we have explored the concept of situated inequality as a theoretical contribution to inequality research (Højholt, 2016, 2022; Højholt & Mardahl-Hansen, 2021; Højholt & Røn Larsen, 2021). As our focus in this article is on how teacher professionalism can be conceptualised in a way that accommodates the complexities and dynamic interactions of everyday life, we will not elaborate on the many discussions of the concept of inequality here.

2 In Denmark, more and more systems have been introduced to assess children—red-yellow-green refers to an assessment of whether everything is okay, fair or alarming.

3 Analyses of the practice example are unfolded in Mardahl-Hansen (2018b), here they primarily support the presentation of the article’s theoretical perspective on situated professionalism.
